# A Blockchain and IPFS-Based Anticounterfeit Traceable Functionality of Car Insurance Claims System

**DOI:** 10.3390/s23239577

**Published:** 2023-12-02

**Authors:** Chin-Ling Chen, Ying-Ming Zheng, Der-Chen Huang, Ling-Chun Liu, Hsing-Chung Chen

**Affiliations:** 1 School of Information Engineering, Changchun Sci-Tech University, Changchun 130600, China; clc@mail.cyut.edu.tw; 2 Department of Computer Science and Information Engineering, Chaoyang University of Technology, Taichung 41349, Taiwan; s11114619@gm.cyut.edu.tw; 3 Department of Computer Science and Engineering, National Chung-Hsing University, Taichung 402202, Taiwan; huangdc@nchu.edu.tw; 4 Department of Computer Science and Information Engineering, Asia University, Taichung 413305, Taiwan; 5 Department of Medical Research, China Medical University Hospital, China Medical University, Taichung 404328, Taiwan

**Keywords:** blockchain, car insurance, Interplanetary File System (IPFS), Hyperledger Fabric, Elliptic Curve Digital Signature Algorithm (ECDSA), smart contract

## Abstract

Due to frequent traffic accidents around the world, people often take out car insurance to mitigate their losses and receive compensation in a traffic accident. However, in the existing car insurance claims process, there are problems such as insurance fraud, inability to effectively track and transmit insurance data, cumbersome insurance procedures, and high insurance data storage costs. Since the immutability and traceability features of blockchain technology can prevent data manipulation and trace past data, we have used the Elliptic Curve Digital Signature Algorithm (ECDSA) to sign and encrypt car insurance data, ensuring both data integrity and security. We propose a blockchain and IPFS-based anticounterfeiting and traceable car insurance claims system to improve the above problems. We incorporate the Interplanetary File System (IPFS) to reduce the cost of storing insurance data. This study also attempts to propose an arbitration mechanism in the event of a car insurance dispute.

## 1. Introduction

Traffic accidents cause driving injuries and property damage. According to World Health Organization (WHO) statistics, about 1.35 million people die in road traffic accidents each year [1], and road traffic accidents are currently estimated to be the eighth leading cause of death in all age groups globally. In addition to the millions of fatalities, the number of traffic accidents in each country is also quite large. In 2020, according to statistics from the National Highway Traffic Safety Administration (NHTSA), US police reported 5,250,837 traffic accidents [2]. The China Statistical Yearbook published by the National Bureau of Statistics of China reported 244,674 traffic accidents [3]. The data in the European Commission’s annual statistical report on road safety are estimated at 757,566 traffic accidents [4]. From the above data, it can be seen that traffic accidents are very dangerous to the global community. Because traffic accidents often require compensation for high medical bills and vehicle repair costs, people will need car insurance to protect themselves and reduce the burden of compensation.

At present, there are still many problems with car insurance. The first is that insurance fraud often occurs between policyholders and insurance companies during the insurance process, such as some policyholders obtaining insurance premiums by forging or falsifying information, and unscrupulous insurance officers forging policies to obtain insurance premiums from policyholders. Car insurance fraud is estimated to cause $29 billion in losses each year [5], and these problems lead to distrust between the public and insurance companies. The second is that in the traditional insurance process, the insurance company has to assign an insurance officer to go out and negotiate with the policyholder on the insurance documents, which will result in the insurance company settling the claim at a much slower speed. When applying for insurance, policyholders often need to bring a lot of documents and spend time meeting and engaging in discussions with insurance personnel, which makes them perceive the process of dealing with insurance matters as troublesome and complicated, and thus they are reluctant to apply for insurance. Third, the insurance company has to bring the application documents and the insured’s information to the medical institution to confirm the insured’s identity when accessing the insured’s medical records as well as to the police agency to obtain authorization for the insured’s traffic accident-related information, which makes the process of obtaining the insured’s information cumbersome and reduces the speed of the claim settlement. All of these problems have created problems for both the insurance company and the policyholder, so it is very important to address them.

To address the challenges mentioned above, a multitude of research efforts have been dedicated to combating car insurance fraud. Notable contributions have been made by Vandervorst et al. [6] and Faheem et al. [7], who employed sophisticated machine learning techniques for the detection of car insurance fraud. Vandervorst et al. focused on evaluating data misrepresentation risk: the insurance company can improve their decision-making process in the validation of insurance contracts with potentially misrepresented self-reported information. Faheem et al. focused on the Boruta algorithm, enabling insurance companies to achieve more effective fraud detection, identification, and examination. Additionally, Shaikh et al. [8] delved into the application of telematics, showcasing its potential to not only reduce instances of car insurance fraud but also improve accident investigation procedures.

Compared to traditional paper insurance documents and the need to repeatedly go out to negotiate, in recent years, many insurance companies have started to use online insurance to make insurance claims. The policyholder only needs to register on the insurance company’s website and can directly start the insurance policy. This form makes the insurance process more convenient by reducing the time it takes the policyholder and the insurance company to process the insurance information and by reducing the generation of paper documents [9].

Although these practices have improved and optimized the car insurance claims process, there are still some unresolved issues. There is still a risk that policy information may be tampered with by the policyholder or the insurance company, and the insurance information may not be traceable. In the existing online insurance claims system, since the insurance information is stored in the insurance company, if the endpoint of the insurance company was to be attacked, the insurance information would likely be stolen or disappear. The policy information and related documents of the online insurance system are also stored in the insurance company, and a large amount of insurance information will cause the insurance company to pay high data storage costs. Additionally, the interaction between insurance companies and medical and police agencies is still weak, and there is no good way to quickly perform functions such as information sharing and transmission for all three parties.

For these reasons, this study uses blockchain technology to solve the above problems. Blockchain is a type of distributed ledger technology: it includes features such as decentralization, anonymity, traceability, and immutability. Decentralization will spread data across many nodes for storage instead of storing them in a single center, thus preventing centralized data control and allowing users on each node to enjoy the same rights. Anonymity is guaranteed since users on the blockchain use a string of English words and numbers as their names, thus protecting their privacy. Traceability is achieved because each block of data on the blockchain contains the hash value of the previous block of data so that the data in the blockchain can be traced back. Immutability means that once the data have been uploaded, they cannot be corrected. Blockchain technology can also be applied to many fields, including financial services [10], manufacturing [11], logistics management [12], agricultural products [13], and insurance services [14,15]. The introduction of blockchain technology can effectively improve the insurance process, improve the security of policy information, increase the efficiency of insurance claims, and reduce the problem of insurance disputes.

This study proposes an anticounterfeit traceable car insurance claims system through blockchain technology with IPFS and Hyperledger Fabric, and aims to achieve the following research objectives:

(1) Immutability: For the security of the insurance data, the data are encrypted, using the hashing operation and uploading the data to the blockchain center to ensure that the data are not tampered with.
(2) Decentralized data sharing: In this paper, we use a Hyperledger Fabric to form a consortium chain among insurance companies, medical institutions, and police agencies. If they want to exchange policyholder information, they can do so securely and efficiently through a Hyperledger Fabric mechanism.
(3) Traceability: Blockchain technology allows insurance data to be uploaded and stored on the blockchain. If the policyholder or insurance company wants to trace insurance data or past medical information, they can go to the blockchain center and IPFS to compare the data and trace the data.
(4) Non-repudiation: Due to the use of the elliptic digital signature algorithm, the data cannot be reversed after it has been transferred to the blockchain, thus providing non-repudiation of the data transfer.
(5) Resist man-in-the-middle attacks: To prevent data transmission from being tampered with by malicious people and forming man-in-the-middle attacks, this paper uses digital signatures and asymmetric encryption techniques to protect against them [16,17].
(6) Reduce storage costs: This article uses IPFS to store some of the data and images generated during the insurance process. Since IPFS is a decentralized storage system, it can reduce data storage costs and permanently store insurance data.

The organization of the rest of the study is as follows. Section 2 will introduce the technical knowledge used in this study, Section 3 will introduce the method and framework used in this study, Section 4 will analyze the security of the method; and Section 5 will compare the calculation and communication costs and finally conclude this study.

## 2. Preliminary

### 2.1. Types and Differences of Blockchains

Blockchain can be divided into three main types: consortium chain, private chain, and public chain [18]:

1. Consortium chain: Consortium chains are typically made up of multiple entities or organizations, and participants must undergo identity verification to join. Consortium chains often have a higher degree of control, as participants can influence and manage the entire blockchain system through consensus mechanisms. Furthermore, consortium chains generally exhibit higher levels of privacy, as only authorized participants have access to and can modify data within the blockchain.
2. Private chain: Private chains are typically controlled by a single organization or enterprise, and only specific participants can join. Private chains usually have a higher degree of control, as the authority to control the blockchain is often held by a single entity or enterprise. Additionally, private chains generally exhibit higher levels of privacy, as only authorized participants have access to and can modify the data within the blockchain.
3. Public chain: Public chains are blockchains that anyone can join, and they operate without centralized control. Public chains usually have lower control because the authority to control the entire blockchain is decentralized among numerous participants. Additionally, public chains typically exhibit greater transparency, as all transactions on the blockchain are public, allowing anyone to view and verify transactions.

The choice between consortium chain, private chain, and public chain depends on the use case and requirements. In the context of this study, consortium chains are used as the framework. The study establishes a consortium chain involving an insurance company, a police agency, and a medical institution, facilitating the sharing of car insurance data among these three parties.

### 2.2. Hyperledger Fabric

Hyperledger Fabric [19] is a blockchain-based distributed ledger framework proposed by the Linux Foundation in 2015. Hyperledger Fabric provides a modular structure which mainly contains an orderer, peer, chaincode, certificate authorities, channel, etc. The orderer is a sorting node which sorts transactions and sends them to a peer. The peer can be part of the chaincode programming language, where smart contracts are implemented through chaincode. The certificate authorities provide a verifiable digital identity for each person who needs to interact with the network. The channel provides a channel for groups to interact with each other internally.

### 2.3. Elliptic Curve Digital Signature Algorithm (ECDSA)

The Elliptic Curve Digital Signature Algorithm was proposed by Don et al. in 2001 [20] as a cryptographic algorithm combining Elliptic Curve Cryptography (ECC) and the Digital Signature Algorithm (DSA). Compared to RSA, ECDSA has higher security than RSA with the same key length. In addition, ECDSA’s small computation size, fast processing speed, and small storage space are all superior to RSA.

The following are the parameters defined in the ECDSA section:

(r,s): Elliptic curve signature value;

(x,y): Characteristic value of the elliptic curve;

h(.): Hash function;

m: Message to be signed;

z: The signature message after a hash;

k: A random number to calculate the values of *r* and *s*;

G: Generating points on an elliptic curve;

$\left(d_{A},Q_{A}\right)$: Private and public key;

$A\overset{?}{=}B$: Verify whether *A* is equal to *B*.

The following is the ECDSA signature and validation process:

(1) Signature phase: First, we need to obtain a curve parameter n and generate a random number k between [1,n−1]; then we calculate:

(1) (x,y)=kG,

(2) z=h(m),

(3) r=xmodn,

(4) $$s=k^{-1}(z+r\times d_{A})\mathrm{mod}n,$$

Finally, obtain the signature result (r,s).

(2) Validation phase: Verify the values of r and s as well as whether $Q_{A}$ and z match. First, verify that (r,s) is between [1,n−1]; then, calculate the following parameters:

(5) z=h(m),

(6) $$u_{1}=z\times s^{-1}\mathrm{mod}n,$$

(7) $$u_{2}=r\times s^{-1}\mathrm{mod}n,$$

(8) $$(x,y)=u1\times G+u2\times Q_{A},$$

Finally, verify:

(9) $$r\overset{?}{=}x\mathrm{mod}n,$$

If the two values are equal, then the signature and the sent message are correct.

### 2.4. Interplanetary File System (IPFS)

IPFS is a system designed by Juan Benet in 2014 [21] to implement a network transfer protocol for the decentralized storage, sharing, and persistence of files. Large data stored on the blockchain can be transferred to IPFS to save storage costs. Files stored on IPFS are hashed to form a file address, and the file can be found by the hash value when accessed [22].

## 3. Method

### 3.1. System Architecture

In this paper, we propose a new car accident insurance claims system using blockchain technology. The system architecture consists of nine main actors:

(1) The policyholder (PH): People who are insured by an insurance company will receive a claim after a traffic accident.
(2) Insurance Company (IC): Insurance companies are responsible for handling insurance claims. After a traffic accident, they will obtain information about the accident from police agencies and medical institutions and notify the bank of the claim.
(3) Police Agency (PA): After the traffic accident, the police will go to the accident site to take photos and identify the traffic accident, which will be sent to the insurance company for processing claims.
(4) Medical Institution (MI): After the traffic accident, the medical institution will make a medical diagnosis of the insured person, and the diagnostic documents will be submitted to the insurance company after the authorization of the medical institution.
(5) Bank (BK): After the accident, the insurance company will use a bank to pay the policyholder’s claim.
(6) Blockchain Center (BCC): All actors in the system need to register with the blockchain center. The blockchain center also receives data files for upload and storage.
(7) Interplanetary File System (IPFS): Images uploaded to the blockchain, such as photos uploaded by PA, medical receipts, diagnoses uploaded by MI, etc., are transferred to IPFS.
(8) Certificate Authorities (CA): Certificate authorities are used to issue personal identity IDs and public and private keys for each character after registration.
(9) Arbitration Institution (AI): Any insurance claim dispute between the actors can be appealed to the arbitration institution.

The seamless functioning of the proposed car accident insurance claim system relies on the collaborative efforts of its various actors. The interactions among the PH, IC, PA, MI, BK, BCC, IPFS, CA, and AI form the foundation of a dynamic and interconnected network. As we enter the distinct phases of the system’s operation, a closer look at the registration, insurance purchasing, traffic accident, claims processing, claims payment, and insurance dispute arbitration phases will illuminate the intricate collaboration and information exchange among these key actors. This collaborative ecosystem ensures the integrity and transparency of the insurance claim process, setting the stage for a more detailed exploration in subsequent sections.

**Figure 1:**
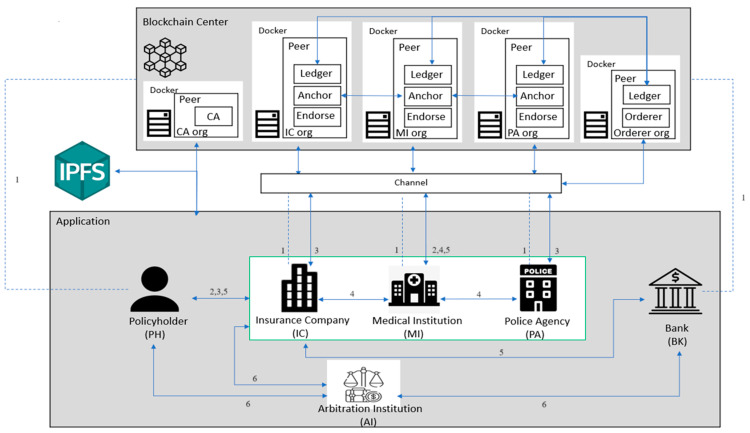
System architecture.

Figure 1 shows the main operational structure of the system. Among this figure, a Ledger is a public database that records all transactions. Anchor typically refers to the process in a transaction in which participants confirm or acknowledge the transaction. Endorse typically refers to the process in a transaction where participants confirm or acknowledge the transaction. The following is a description of each phase.

(1) Registration Phase: The relevant actors in the system need to apply for an account with the BCC, and after confirmation, they will upload the account information to the BCC. The CA will issue the corresponding public key and private key. In this case, the MI administrator will organize the members of the MI, the PA, and the IC into a consortium chain to pass information about the insured to each other.
(2) Insurance Purchasing Phase: After the PH purchases insurance from the IC, the IC and the PH will sign an insurance policy. The PH must provide the bank account to the IC. After signing the insurance policy, it is uploaded to the BCC, and the policy ID is submitted to the PH.
(3) Traffic Accident Phase: When the PH is involved in a traffic accident, the PH should first notify the PA to deal with the accident. After the PA arrives at the scene, the PA will take photos to confirm the traffic accident, make a record of the accident, and finally upload the photos and identity records to the BCC. The medical receipts and diagnoses of the PH will be uploaded from the MI to the blockchain center.
(4) Claims Processing Phase: When the PH submits a claim, the IC will request authorization of the PH’s medical receipt information of the PH from the MI where the PH was treated through the PH’s ID. After authorization, the claim will be evaluated in conjunction with the accident identification records provided by the PA.
(5) Claims Payment Phase: The IC notifies the BK to pay the PH’s claim and then uploads the claim record to the BCC and notifies the PH.
(6) Insurance Dispute Arbitration Phase: When an IC denies a claim or when there is an insurance dispute between the parties involved in the traffic accident, arbitration can be conducted through AI.

### 3.2. Registration Phase

In the registration phase, all roles must first register with the BCC first. 

Step 1: Role X first generates an identity, $ID_{X}$, and sends $ID_{X}$ to the BCC.

The BCC generates an ECDSA private key $d_{X}$ and calculates the public key $Q_{X}$ of role *X*:

(10) $$Q_{X}=d_{X}\times G,$$

Step 2: The BCC sends $ID_{X},(Q_{X},d_{X})$ back to role *X*, and role *X* stores the $\left(Q_{X},d_{X}\right)$.

### 3.3. Insurance Purchasing Phase

When the PH purchases car insurance from the IC, the IC verifies the identity of the PH and signs a policy. The PH will provide the IC with their bank account and then send the generated CC and CD to the BCC. 

Step 1: The IC first gives the PH an insurance policy $M_{\det ail}$. The PH will first confirm the content of the policy. After confirmation, the PH will confirm and complete the basic insurance information in the policy to form ${M_{\det ail}}^{\prime }$; then, the PH selects a random value $K_{PH-IC}$ and uses the IC’s public key $PUK_{IC}$ for encryption:

(11) $$C_{PH-IC}=E_{PUK_{IC}}({M_{\det ail}}^{\prime })$$

Then, calculate the ECDSA parameters:

(12) $$token=h(ID_{PH},{M_{\det ail}}^{\prime },TS_{PH-IC})$$

Pass the parameter $\left(token,k_{PH-IC},d_{PH}\right)$ to the sign function of Algorithm 1 to obtain the signature value $\left(r_{PH-IC},s_{PH-IC}\right)$. Finally, the PH sends $ID_{PH},C_{PH-IC},$$(r_{PH-IC},s_{PH-IC}),\mathrm{timestamp},$ $TS_{PH-IC}$ to the IC.

Step 2: The IC will check the validity of the timestamp after receiving the encrypted information from the PH:

(13) $$TS_{NOW}-TS_{PH-IC}\leq \Delta T$$

Use the IC’s private key to decrypt the encrypted data and obtain the content:

(14) $${M_{\det ail}}^{\prime }=D_{PRK_{IC}}{(C}_{PH-IC})$$

Then, calculate the ECDSA parameters:

(15) $${token}^{\prime }=h(ID_{PH},{M_{\det ail}}^{\prime },TS_{PH-IC})$$

Pass the parameter $\left({token}^{\prime },(r_{PH-IC},s_{PH-IC}),Q_{PH}\right)$ to the verify function of Algorithm 2 to verify whether $r_{PH-IC}$ is correct. If it is correct, the IC will obtain the PH’s information. Generate $M_{Policy}$ from $(ID_{PH},ID_{IC},$ ${M_{\det ail}}^{\prime }$$,TS_{PH-IC})$. Then, generate a claim certificate $CC_{PH}$ and claim detail $CD_{PH}$. Finally, upload the policy identity $ID_{Policy}$ and $(BC_{PH-IC},CC_{PH},$$CD_{PH}$$,M_{Policy})$ to the blockchain.

After uploading the information, to allow the PH to view his insurance information, the IC will select a random value $k_{IC-PH}$ and use the PH’s public key $PUK_{PH}$ to encrypt $M_{Policy}$:

(16) $$C_{IC-PH}=E_{PUK_{PH}}(M_{Policy})$$

Then, calculate the ECDSA parameters:

(17) $$token=h(ID_{PA},C_{IC-PH},TS_{IC-PH})$$

Pass the parameter $\left(token,k_{IC-PH},d_{PA}\right)$ to the Sign function of Algorithm 1 to obtain the signature value $(r_{IC-PH},$$s_{IC-PH})$, which the IC sends $ID_{IC},C_{IC-PH},$ $(r_{IC-PH},s_{IC-PH}),TS_{IC-PH}$ to the PH.

Step 3: The PH will check the validity of the timestamp after receiving the encrypted information from the IC:

(18) $$TS_{NOW}-TS_{IC-PH}\leq \Delta T$$

Use the PH’s private key to decrypt the encrypted data and obtain the content:

(19) $$M_{IC-PH}=D_{PRK_{PH}}(C_{IC-PH})$$

Then, calculate the ECDSA parameters:

(20) $${token}^{\prime }=h(ID_{IC},C_{IC-PH},TS_{IC-PH})$$

Pass the parameter $\left({token}^{\prime },r_{IC-PH},s_{IC-PH},Q_{IC}\right)$ to the verify function of Algorithm 2 to verify whether $r_{IC-PH}$ is correct; if it is correct, the PH will be able to view his insurance information.

**Algorithm 1** Function sign(token,k,d)
   z=token;   (x,y)=kG;   r=xmodn;   $s=k^{-1}(z+rd)\mathrm{mod}n$;return (r,s);

**Algorithm 2** Function verify(token,r,s,Q)   $z^{\prime }=token$;   $u_{{}_{1}}=z^{\prime }\times s^{-1}\mathrm{mod}n$;   $u_{{}_{2}}=r\times s^{-1}\mathrm{mod}n$;   
$(x^{\prime },y^{\prime })=u_{{}_{1}}G+u_{{}_{2}}Q$
   
$r\overset{?}{=}x^{\prime }\mathrm{mod}n$
return *true/false*;

### 3.4. Traffic Accident Phase

After a traffic accident, the PH will notify the PA of the accident. The PH will provide the PA with his ID, complete the accident registration form and his public key, and provide his driver’s license and vehicle registration to the PA for verification. The PA will take photos of the accident and make an accident identification record. The photos and identification will then be stored in IPFS, and the stored data will be hashed and uploaded to the blockchain with the PH’s ID.

Step 1: The PH first fills out a traffic accident registration form, which is also filled out for the other owner of the vehicle involved in the accident, generating $M_{PH-OS-PA}$. Then, the PH selects a random value $k_{PH-PA}$ and uses the IC’s public key $PUK_{PA}$ for encryption:

(21) $$C_{PH-PA}=E_{PUK_{PA}}(M_{PH-OS-PA})$$

Then, calculate the ECDSA parameters:

(22) $$token=h(ID_{PH},C_{PH-PA},TS_{PH-PA})$$

Pass the parameter $\left(token,k_{PH-PA},d_{PH}\right)$ to the Sign function of Algorithm 1 to obtain the signature value $\left(r_{PH-PA},s_{PH-PA}\right)$, which the PH sends $ID_{PH},C_{PH-PA},$$(r_{PH-PA},s_{PH-PA}),TS_{PH-PA}$ to the PA.

Step 2: The PA will verify the validity of the timestamp after receiving the encrypted information from the PH:

(23) $$TS_{NOW}-TS_{PH-PA}\leq \Delta T$$

Use the PA’s private key to decrypt the encrypted data and obtain the content:

(24) $$M_{PH-OS-PA}=D_{PRK_{PA}}(C_{PH-PA})$$

Then, calculate the ECDSA parameters:

(25) $${token}^{\prime }=h(ID_{PH},C_{PH-PA},TS_{PH-PA})$$

Pass the parameter $\left({token}^{\prime },r_{PH-PA},s_{PH-PA},Q_{PH}\right)$ to the Verify function of Algorithm 2 to verify whether $r_{PH-PA}$ is correct; if it is correct, the PA will obtain the PH’s information and record the traffic accident identity $ID_{TA}$. After that, the PA saves the photos of the accident $M_{Picture}$ and the traffic accident identification records to IPFS and obtains the hashed photos of the accident $PIC_{PH}$ and the accident identification certificate $IDE_{PH}$. Finally, upload $\left(ID_{TA},BC_{PH-PA},PIC_{PH},IDE_{PH},TA_{ID}\right)$ to the blockchain center. After uploading the information, to allow the PH to view his insurance information, the PA will generate the message $M_{PA-PH}$ with the relevant information, select a random value $k_{PA-PH}$, and use the PH’s public key $PUK_{PH}$ for encryption:

(26) $$C_{PA-PH}=E_{PUK_{PH}}(M_{PA-PH})$$

Then, calculate the ECDSA parameters:

(27) $$token=h(ID_{PA},C_{PA-PH},TS_{PA-PH})$$

Pass the parameter $\left(token,k_{PA-PH},d_{PA}\right)$ to the Sign function of Algorithm 1 to obtain the signature value $\left(r_{PA-PH},s_{PA-PH}\right)$; then, the PA sends $ID_{PA},C_{PA-PH},$$(r_{PA-PH},s_{PA-PH}),TS_{PA-PH}$ to PH.

Step 3: The PH will verify the validity of the timestamp after receiving the encrypted information from the PA:

(28) $$TS_{NOW}-TS_{PA-PH}\leq \Delta T$$

Use the PH’s private key to decrypt the encrypted data and obtain the content:

(29) $$M_{PA-PH}=D_{PRK_{PH}}(C_{PA-PH})$$

Then, calculate the ECDSA parameters:

(30) $${token}^{\prime }=h(ID_{PA},C_{PA-PH},TS_{PA-PH})$$

Pass the parameter $\left({token}^{\prime },r_{PA-PH},s_{PA-PH},Q_{PA}\right)$ to the Verify function of Algorithm 2 to verify whether it is correct. If it is correct, then the PH can view his traffic accident-related information.

### 3.5. Medical Phase

After filling out the traffic accident information, the PH will go to the MI to seek medical treatment. The PH will need to provide his ID and public key to the MI and fill in basic personal information. Medical receipts and diagnoses will be stored in IPFS by the MI, and the stored data will be washed and uploaded to the blockchain center with the PH’s ID.

Step 1: The PH first fills out basic personal information and generates $M_{PH-MI}$; then, the PH selects a random value $k_{PH-MI}$ and uses the MI’s public key $PUK_{MI}$ for encryption:

(31) $$C_{PH-MI}=E_{PUK_{MI}}(M_{PH-MI})$$

Then, calculate the ECDSA parameters:

(32) $$token=h(ID_{PH},C_{PH-MI},TS_{PH-MI})$$

Pass the parameter $\left(token,k_{PH-MI},d_{PH}\right)$ to the Sign function of Algorithm 1 to obtain the signature value $\left(r_{PH-MI},s_{PH-MI}\right)$, which the PH sends $ID_{PH},C_{PH-MI},$$(r_{PH-MI},s_{PH-MI}),TS_{PH-MI}$ to the MI.

Step 2: The MI will check the validity of the timestamp after receiving the encrypted information from the PH:

(33) $$TS_{NOW}-TS_{PH-MI}\leq \Delta T$$

The MI’s private key is used to decrypt the encrypted data and obtain the content:

(34) $$M_{PH-MI}=D_{PRK_{MI}}(C_{PH-MI})$$

Then, calculate the ECDSA parameters:

(35) $${token}^{\prime }=h(ID_{PH},C_{PH-MI},TS_{PH-MI})$$

Pass the parameter $\left({token}^{\prime },r_{PH-MI},s_{PH-MI},Q_{PH}\right)$ to the Verify function of Algorithm 2 to verify whether $r_{PH-MI}$ is correct; if it is correct, the MI will obtain the PH’s information and save the PH’s medical diagnosis and medical receipt data to IPFS, and then it will obtain the hashed diagnosis $CERT_{PH}$ and receipts $REC_{PH}$. Finally, the PH uploads the $(ID_{TA},$ $BC_{PH-MI},$ $CERT_{PH},$ $REC_{PH})$ to the blockchain. After the PH uploads the information, to allow the PH to view his insurance information, the MI will generate the message $M_{MI-PH}$ with the relevant information, select a random value $k_{MI-PH}$, and use the PH’s public key $PUK_{PH}$ for encryption:

(36) $$C_{MI-PH}=E_{PUK_{PH}}(M_{MI-PH})$$

Then, calculate the ECDSA parameters:

(37) $$token=h(ID_{MI},C_{MI-PH},TS_{MI-PH})$$

Pass the parameter $\left(token,k_{MI-PH},d_{PA}\right)$ to the Sign function of Algorithm 1 to obtain the signature value $\left(r_{MI-PH},s_{MI-PH}\right)$, which the MI sends $ID_{MI},C_{MI-PH},$$(r_{MI-PH},s_{MI-PH}),TS_{MI-PH}$ to the PH.

Step 3: The PH will check the validity of the timestamp after receiving the encrypted information from the MI:

(38) $$TS_{NOW}-TS_{MI-PH}\leq \Delta T$$

Use the PH’s private key to decrypt the encrypted data and obtain the content:

(39) $$M_{MI-PH}=D_{PRK_{PH}}(C_{MI-PH})$$

Then, calculate the ECDSA parameters:

(40) $${token}^{\prime }=h(ID_{MI},C_{MI-PH},TS_{MI-PH})$$

Pass the parameter $\left({token}^{\prime },r_{MI-PH},s_{MI-PH},Q_{MI}\right)$ to the Verify function of Algorithm 2 to verify whether it is correct; if it is correct, then the PH can view his medical information.

### 3.6. Claims Processing Phase

When the PH has received the relevant documents from the PH and MI, he can notify the IC to proceed with the claim processing. The PH will authorize the IC to have access to the PH’s information and to process the claim.

#### 3.6.1. Request for Authorization of the Traffic Accident Identification Process

After the PA has processed the PH’s accident identification data, the PH can notify the IC to proceed with claim processing. The IC will submit the relevant policyholder’s claim documents and its ID to the PA to identify itself. After the PA agrees to authorize the information, the IC can obtain the policyholder’s information regarding the identification of the traffic accident.

Step 1: The PH submits his identity $ID_{PH}$ and traffic accident identity $TK_{ID}$ to the IC for authorization. Once the information is available, the IC will provide the data access to the PA.

Step 2: The IC will send a message $M_{IC-PA}$ to the PA with the identity of the IC and the policy identity, claim certificate, claim details, ID, and traffic accident identity of the PH. The system encrypts the data content and generates $C_{IC-PA}$; then, the IC selects a random value $k_{IC-PA}$ and uses the PA’s public key $PUK_{PA}$ for encryption:

(41) $$C_{IC-PA}=E_{PUK_{PA}}(M_{IC-PA})$$

Then, calculate the ECDSA parameters:

(42) $$token=h(ID_{IC},C_{IC-PA},TS_{IC-PA})$$

Pass the parameter $\left(token,k_{IC-PA},d_{IC}\right)$ to the Sign function of Algorithm 1 to obtain the signature value $\left(r_{IC-PA},s_{IC-PA}\right)$. Finally, the IC sends $ID_{IC},$$C_{IC-PA},$$(r_{IC-PA},s_{IC-PA}),TS_{IC-PA}$ to PA:

Step 3: The PA will check the validity of the timestamp after receiving the encrypted information from the IC:

(43) $$TS_{NOW}-TS_{IC-PA}\leq \Delta T$$

Use the PA’s private key to decrypt the encrypted data and obtain the content:

(44) $$M_{IC-PA}=D_{PRK_{PA}}(C_{IC-PA})$$

Then, calculate the ECDSA parameters:

(45) $${token}^{\prime }=h(ID_{IC},C_{IC-PA},TS_{IC-PA})$$

Pass the parameter $\left({token}^{\prime },r_{IC-PA},s_{IC-PA},Q_{IC}\right)$ to the Verify function of Algorithm 2 to verify whether $r_{IC-PA}$ is correct; if it is correct, the PA will obtain the application information from the IC. Finally, upload the $\left(ID_{IC},BC_{IC-PA}\right)$ to the blockchain. After uploading the information, to allow the IC to view the PH’s traffic accident identification, the PA will generate the message $M_{PA-IC}$ with the relevant information, select a random value $k_{PA-IC}$, and use the IC’s public key $PUK_{IC}$ for encryption:

(46) $$C_{PA-IC}=E_{PUK_{IC}}(M_{PA-IC})$$

Then, calculate the ECDSA parameters:

(47) $$token=h(ID_{PA},C_{PA-IC},TS_{PA-IC})$$

Pass the parameter $\left(token,k_{PA-IC},d_{PA}\right)$ to the Sign function of Algorithm 1 to obtain the signature value $\left(r_{PA-IC},s_{PA-IC}\right)$; then, the PA sends $ID_{PA},C_{PA-IC},$ $(r_{PA-IC},s_{PA-IC}),TS_{PA-IC}$ to IC.

Step 4: The IC will verify the validity of the timestamp after receiving the encrypted information from the PA:

(48) $$TS_{NOW}-TS_{PA-IC}\leq \Delta T$$

Use the IC’s private key to decrypt the encrypted data and obtain the content:

(49) $$M_{PA-IC}=D_{PRK_{IC}}(C_{PA-IC})$$

Then, calculate the ECDSA parameters:

(50) $${token}^{\prime }=h(ID_{PA},C_{PA-IC},TS_{PA-IC})$$

Pass the parameter $\left({token}^{\prime },r_{PA-IC},s_{PA-IC},Q_{PA}\right)$ to the Verify function of Algorithm 2 to verify whether $r_{PA-IC}$ is correct; if it is correct, then the IC can view the information related to the identification of the PH’s traffic accidents.

#### 3.6.2. Request for Authorization of the Medical Diagnostic Information Process

The IC will request authorization for the PH’s medical information from the MI after receiving the traffic accident identification records. Once the MI has authorized the information, the IC will obtain the medical diagnosis and receipts and combine them with the identification records to process the claim.

Step 1: The PH submits his identity $ID_{PH}$ and the identity of the MI $ID_{MI}$ where he was treated at the time of the traffic accident to the IC for authorization; once the information is available, the IC will enable the data access of the MI.

Step 2: The IC will send a message $M_{IC-MI}$ to the MI with the identity of the IC and the policy identity, claim certificate, claim details, and ID. The system encrypts the data content and generates $C_{IC-MI}$; then, the IC selects a random value $k_{IC-MI}$ and uses the MI’s public key $PUK_{MI}$ for encryption:

(51) $$C_{IC-MI}=E_{PUK_{MI}}(M_{IC-MI})$$

Then, calculate the ECDSA parameters:

(52) $$token=h(ID_{IC},C_{IC-MI},TS_{IC-MI})$$

Pass the parameter $\left(token,k_{IC-MI},d_{IC}\right)$ to the Sign function of Algorithm 1 to obtain the signature value $\left(r_{IC-MI},s_{IC-MI}\right)$; then, the IC sends $ID_{IC},C_{IC-MI},$ $(r_{IC-MI},s_{IC-MI}),TS_{IC-MI}$ to MI.

Step 3: The MI will verify the validity of the timestamp after receiving the encrypted information from the IC:

(53) $$TS_{NOW}-TS_{IC-MI}\leq \Delta T$$

Use the MI’s private key to decrypt the encrypted data and obtain the content:

(54) $$M_{IC-MI}=D_{PRK_{MI}}(C_{IC-MI})$$

Then, calculate the ECDSA parameters:

(55) $${token}^{\prime }=h(ID_{IC},C_{IC-MI},TS_{IC-MI})$$

Pass the parameter $\left({token}^{\prime },r_{IC-MI},s_{IC-MI},Q_{IC}\right)$ to the Verify function of Algorithm 2 to verify whether $r_{IC-MI}$ is correct. If it is correct, the MI will obtain the application information from the IC. Finally, upload the $\left(ID_{TA},BC_{IC-MI}\right)$ to the blockchain. After uploading the information, to allow the IC to view the medical diagnosis and medical receipts of the PH, the IC will generate the message $M_{MI-IC}$ with the relevant information, select a random value $k_{MI-IC}$, and use the public key $PUK_{IC}$ for encryption:

(56) $$C_{MI-IC}=E_{PUK_{IC}}(M_{MI-IC})$$

Then, calculate the ECDSA parameters:

(57) $$token=h(ID_{MI},C_{MI-IC},TS_{MI-IC})$$

Pass the parameter $\left(token,k_{MI-IC},d_{MI}\right)$ to the Sign function of Algorithm 1 to obtain the signature value $\left(r_{MI-IC},s_{MI-IC}\right)$; then, the MI sends $ID_{MI},$$C_{MI-IC},$$(r_{MI-IC},s_{MI-IC}),TS_{MI-IC}$ to the IC.

Step 4: The IC will check the validity of the timestamp after receiving the encrypted information from the MI:

(58) $$TS_{NOW}-TS_{MI-IC}\leq \Delta T$$

Use the IC’s private key to decrypt the encrypted data and obtain the content:

(59) $$M_{MI-IC}=D_{PRK_{IC}}(C_{MI-IC})$$

Then, calculate the ECDSA parameters:

(60) $${token}^{\prime }=h(ID_{MI},C_{MI-IC},TS_{MI-IC})$$

Pass the parameter $\left({token}^{\prime },r_{MI-IC},s_{MI-IC},Q_{MI}\right)$ to the Verify function of Algorithm 2 to verify whether $r_{MI-IC}$ is correct; if it is correct, then the IC can view the PH’s medical diagnosis and medical receipts.

### 3.7. Claims Payment Phase

After the IC confirms the claim, they will notify the BK to pay the claim to the PH. The BK will notify the PH of the successful payment.

Step 1: The IC encrypts the PH’s identification ID, bank account ID, and the insurance policy signed by the PH to generate a message $M_{IC-BK}$; then, the IC selects a random value $k_{IC-BK}$ and uses the public key $PUK_{BK}$ for encryption:

(61) $$C_{IC-BK}=E_{PUK_{BK}}(M_{IC-BK})$$

Then, calculate the ECDSA parameters:

(62) $$token=h(ID_{IC},C_{IC-BK},TS_{IC-BK})$$

Pass the parameter $\left(token,k_{IC-BK},d_{IC}\right)$ to the Sign function of Algorithm 1 to obtain the signature value $\left(r_{IC-BK},s_{IC-BK}\right)$; then, the IC sends $ID_{IC},C_{IC-BK}$$,(r_{IC-BK},s_{IC-BK}),TS_{IC-BK}$ to BK.

Step 2: The BK will check the validity of the timestamp after receiving the encrypted information from the IC:

(63) $$TS_{NOW}-TS_{IC-BK}\leq \Delta T$$

Uses the BK’s private key to decrypt the encrypted data and obtain the content:

(64) $$M_{IC-BK}=D_{PRK_{BK}}(C_{IC-BK})$$

Then, calculate the ECDSA parameters:

(65) $${token}^{\prime }=h(ID_{IC},C_{IC-BK},TS_{IC-BK})$$

Pass the parameter $\left({token}^{\prime },r_{IC-BK},s_{IC-BK},Q_{IC}\right)$ to the Verify function of Algorithm 2 to verify whether $r_{IC-BK}$ is correct; if it is correct, the BK will obtain the data and call the smart contract for payment of compensation. After the payment, the BK will upload $\left(ID_{TA},BC_{IC-BK}\right)$ to the blockchain center; then, the BK will send a notification $M_{BK-PH}$ to the PH, and the BK will select a random value $k_{BK-PH}$; then, calculate the ECDSA parameters:

(66) $$token=h(ID_{BK},M_{BK-PH},TS_{BK-PH})$$

Pass the parameter $\left(token,k_{BK-PH},d_{BK}\right)$ to the Sign function of Algorithm 1 to obtain the signature value $\left(r_{BK-PH},s_{BK-PH}\right)$; then, the BK sends $ID_{BK},M_{BK-PH},(r_{BK-PH},s_{BK-PH}),TS_{BK-PH}$ to the PH.

Step 3: The PH will check the validity of the timestamp after receiving the encrypted information from the BK:

(67) $$TS_{NOW}-TS_{BK-PH}\leq \Delta T$$

Then, calculate the ECDSA parameters:

(68) $${token}^{\prime }=h(ID_{BK},M_{BK-PH},TS_{BK-PH})$$

Pass the parameter $\left({token}^{\prime },r_{BK-PH},s_{BK-PH},Q_{BK}\right)$ to the Verify function of Algorithm 2 to verify whether $r_{BK-PH}$ is correct; if it is correct, the PH receives the BK remittance notification and successfully obtains the claim.

### 3.8. Insurance Dispute Arbitration Phase

When a dispute arises between the PH, the IC or the BK, the PH can propose an arbitration application to the AI. The PH will provide related information for the AI to confirm the PH’s information and identity.

**Figure 2:**
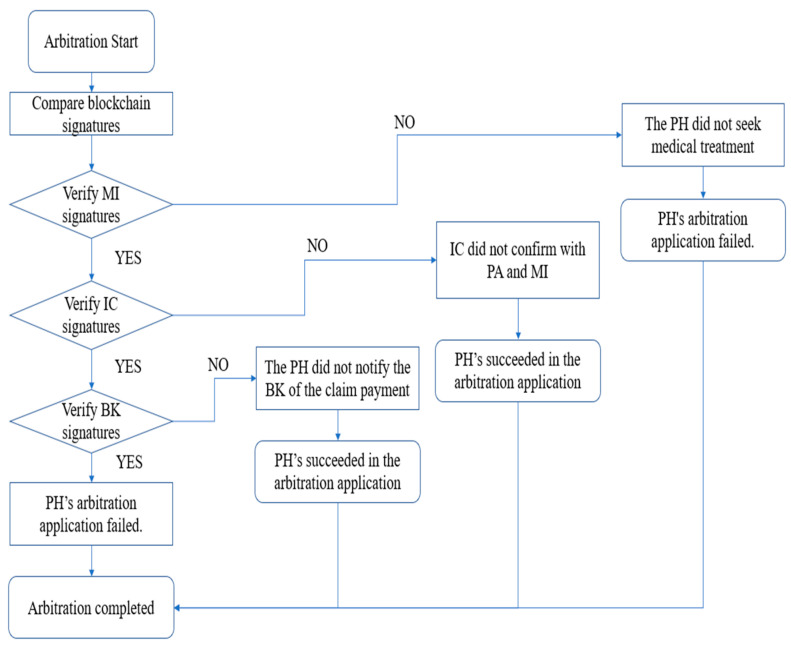
The flow chart during the insurance dispute arbitration phase.

When arbitrating insurance disputes, the AI confirms the accuracy of each phase of operation by comparing the information and signatures on the blockchain. Figure 2 shows the flow chart during the insurance dispute arbitration phase.

Step 1: To confirm the authenticity of the event, the PH must first submit $ID_{PH}$, $ID_{Policy}$, and $ID_{TA}$ to the AI. After confirming receipt of the PH’s documents, the AI will verify them. If the verification fails, it means that the PH has provided incorrect information; otherwise, the verification is successful, and it will begin signing verification.

Step 2: The AI will first pass the PH’s $ID_{TA}$ and obtain the signature values $\left(r_{PH-MI},s_{PH-MI}\right)$, $\left(r_{MI-PH},s_{MI-PH}\right)$, $\left(r_{IC-PA},s_{IC-PA}\right)$, $\left(r_{PA-IC},s_{PA-IC}\right)$, $\left(r_{IC-MI},s_{IC-MI}\right)$, $\left(r_{MI-IC},s_{MI-IC}\right)$, $\left(r_{IC-BK},s_{IC-BK}\right)$, and $\left(r_{BK-PH},s_{BK-PH}\right)$, and then it will verify according to the signature value.

The AI will first verify $BC_{PH-MI}$ and $BC_{MI-PH}$:

(69) $$BC_{PH-MI}\overset{?}{=}h(r_{PH-MI},s_{PH-MI})$$

(70) $$BC_{MI-PH}\overset{?}{=}h(r_{MI-PH},s_{MI-PH})$$

If the verification fails, it means that the PH did not perform the action of medical treatment, and if the verification is successful, the procedure will proceed to the next step.

Step 3: The AI then verifies the signature of the PH and will simultaneously verify $BC_{IC-PA}$, $BC_{PA-IC}$, $BC_{IC-MI}$, and $BC_{MI-IC}$:

(71) $$BC_{IC-PA}\overset{?}{=}h(r_{IC-PA},s_{IC-PA})$$

(72) $$BC_{PA-IC}\overset{?}{=}h(r_{PA-IC},s_{PA-IC})$$

(73) $$BC_{IC-MI}\overset{?}{=}h(r_{IC-MI},s_{IC-MI})$$

(74) $$BC_{MI-IC}\overset{?}{=}h(r_{MI-IC},s_{MI-IC})$$

If one of the verifications fails, it means that the IC has not processed the claim with the PA and MI, and if the verification is successful, the procedure will proceed to the next step.

Step 4: The AI will take the signature of the verifying BK and verify $BC_{IC-BK}$ and $BC_{BK-PH}$:

(75) $$BC_{IC-BK}\overset{?}{=}h(r_{IC-BK},s_{IC-BK})$$

(76) $$BC_{BK-PH}\overset{?}{=}h(r_{BK-PH},s_{BK-PH})$$

If the verification fails, it means that the BK did not proceed with the claim payment.

If all of the above steps have been verified successfully, the AI can determine the validity of the insurance claim and terminate the arbitration procedure.

## 4. Security Analysis

### 4.1. Non-Repudiation

Since ECDSA is used for data signature and verification, each time the data are signed, they must be signed with the sender’s private key, and the receiver also needs the public key for verification. After the data are transmitted to the recipient, this action cannot be reversed, and the receiver can confirm its identity through the public key, thus achieving data non-repudiation. Table 1 shows the non-repudiation in each phase of this study.

### 4.2. Decentralized Data Sharing

In this study, we combine the Hyperledger system, where members of the consortium chain must register before sharing data. The flow of information is open and transparent for all members of the same consortium chain. Information cannot be withheld between nodes of the consortium chain. In this way, the trust relationship between nodes is realized.

The study uses the MI, the PA, and the IC as a consortium chain to enable the three parties to transmit the PH’s information to each other. The need for data transfer and updates on Hyperledger Fabric requires the agreement of other peer nodes, which is a process also known as consensus. When the consensus of the group reaches 66.6% or more, the data will be sorted by order nodes and transferred to the node to complete the data transfer. Through this mechanism, the decentralized data-sharing feature is achieved. 

### 4.3. Traceability

Once the data are uploaded to the blockchain, they cannot be tampered with, and the data on the chain are traceable.

For example, if the PH wants to trace blockchain data during the insurance purchase phase, the PH can verify it by comparing $BC_{PH-IC}=h(r_{PH-IC},s_{PH-IC})$ and $BC_{IC-PH}=h(r_{IC-PH},s_{IC-PH})$. If the PH wants to trace the blockchain data during the traffic accident phase, they can verify it by comparing $BC_{PH-PA}=h(r_{PH-PA},s_{PH-PA})$ and $BC_{PA-PH}=h(r_{PA-PH},s_{PA-PH})$. If the PH wants to trace the blockchain data during the medical phase, they can verify it by comparing $BC_{PH-MI}=h(r_{PH-MI},s_{PH-MI})$ and $BC_{MI-PH}=h(r_{MI-PH},s_{MI-PH})$. Through the above matching method, we can ensure the traceability of data.

### 4.4. Immutability 

During the signature phase, the sender encrypts the message to be transmitted and adds it to the signature program to obtain the signature value to be transmitted to the receiver. If the receiver finds that the comparison is unsuccessful during data validation, it means that the value of the hash function may have been tampered with and the transmitted message is not the original in order to prevent data tampering. Table 2 shows the data manipulation prevention components in each phase of this document.

### 4.5. Man-in-the-Middle Attacks

To prevent malicious people from altering the content of the data during data transmission and forming a man-in-the-middle attack, this study uses digital signatures and asymmetric cryptography to defend against the attack. When the sender transmits data, the public key can be used to encrypt the data content, and the receiver can use the private key to decrypt the data after receiving them. In this study, the public key is used to encrypt the data, and middlemen cannot obtain the data and manipulate it, so the data can be protected from attack. Table 3 shows the man-in-the-middle attack defense in each phase of this study.

## 5. Discussions

### 5.1. Computation Cost

Table 4 presents the computational cost analysis for each phase of this study, which summarizes the computational time cost of signature verification, function judgment, hashing, and multiplication for each phase.

### 5.2. Communication Cost

Table 5 shows the total communication cost for each stage of the study. For example, the insurance purchasing phase contains two times ECDSA encryption, two times asymmetric encryption, two times IPFS file hash value, four times ID and other information in the transmission of the message, and the total message length is as follows:

2 × 1024 bits + 2 × 512 bits + 2 × 368 bits + 4 × 80 bits = 4128 bits

In a 4G communication environment, the time taken for the insured phase is 0.04 ms, while in a 5G communication environment, it takes 0.2 µs to demonstrate the communication speed of each phase.

### 5.3. Performance Analysis

In this section, a reasonable blockchain chain code performance evaluation test will be conducted on the processes in this study. The testing tool is Hyperledger Caliper version 0.5.0, and the runtime environment used for Caliper is Node.js version 16. The blockchain platform used is Hyperledger Fabric version 2.2. The server performance is Core i9 9920X@4.5Ghz CPU and 24 GB RAM, and the operating system is Ubuntu 20.04.

First, the required data and photos will be stored in IPFS. After storing the data in IPFS, the IPFS ID is obtained. Then, we obtain the blockchain address of the data stored in the blockchain; the IPFS ID and blockchain address are written into a chaincode. We configure the environment required for Hyperledger Fabric and Caliper and set up the various node organizations. Each node organization joins the chaincode for performance testing. The throughput on the blockchain represents the TPS (transactions per second) that can be loaded on the system, and the transaction latency is the time it takes from the time a transaction is initiated to the time a confirmation is received that the transaction is valid.

**Figure 3:**
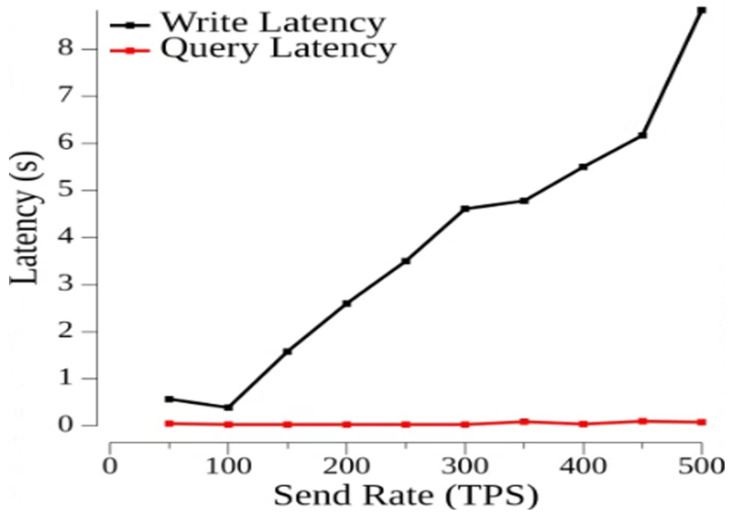
Latency time in different workloads.

**Figure 4:**
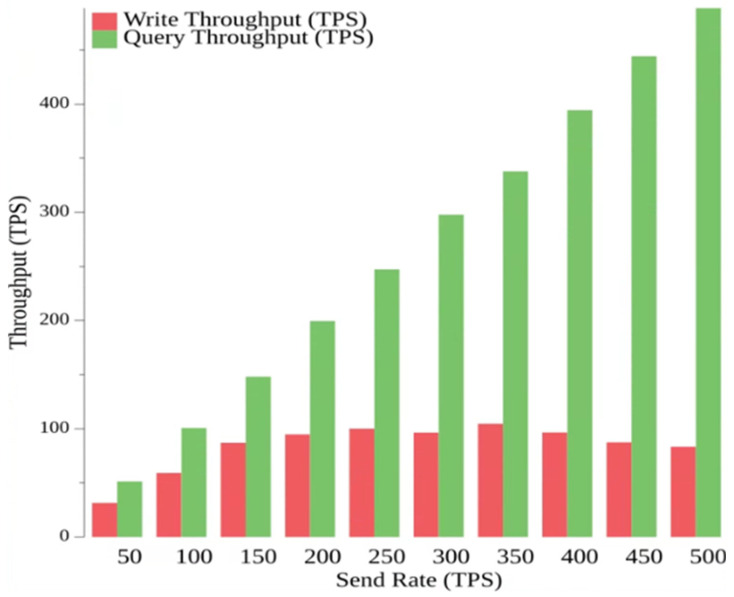
Throughput in different workloads.

In this test, we chose 10 sending rate groups from 50 to 500 tps and used load balancing to optimize CPU usage to reduce the server load with a transaction duration of 3 s and several clients of 6. In Figure 3, we analyze the relationship between sending rate and transaction latency. The minimum delay in writing data is 0.42 s, and the maximum delay is 7.13 s. The minimum delay for writing data is 0.42 s, and the maximum delay is 7.13 s. The minimum delay for querying data is 0.01 s, and the maximum delay is 0.02 s. Thus, it can be seen that the latency of the write phase will increase with the sending rate when the sending interval is a certain size, while the latency of the read phase remains stable. In Figure 4, the relationship between the transmit rate and throughput is analyzed. The minimum throughput of a write is 31.8 tps and the maximum is 112.8 tps, while the minimum throughput of a read is 51.1 tps and the maximum is 491 tps.

From the above tests, it can be concluded that the proposed claims system can store and read a large amount of data in the actual insurance data, and the consortium chain members in the system can effectively store the policyholder data on the blockchain and can quickly access the data and interact with other parties if they want to access the data.

### 5.4. Comparison Table of the Characteristics of Related Work

In this section, we compare our proposed solution with other articles on the application of blockchain in insurance in Table 6.

## 6. Conclusions

The study used Hyperledger Fabric combined with IPFS for system performance analysis. In the experimental environment, the system demonstrated a minimum throughput of 31.8 tps and a maximum throughput of 112.8 tps for write operations. For read operations, the minimum throughput was 51.1 tps and the maximum throughput reached 491 tps, confirming the feasibility of the system performance. Furthermore, the latency time for write operations was recorded at 7.13 s, while read operations had a latency time of 0.02 s, both falling within acceptable time ranges. Such insurance data, whether in terms of latency time or throughput, can provide efficient transmission results for insurance data. Additionally, blockchain technology ensures the anticounterfeit traceable functionality of insurance data. The system also incorporates ECDSA encryption technology, achieving the goal of non-repudiation.

For any insurance company, this study can reduce the time cost of meeting with the policyholder, increase the speed of claim settlement, improve corporate reputation, and quickly obtain relevant authorization information from police agencies and medical institutions. For the policyholder, it can save the complicated claim application procedure and reduce the time required for insurance, and because the insurance documents and traffic accident information are stored on the blockchain and IPFS, the policyholder does not need to carry a lot of insurance documents in the process of applying for insurance claims.

## Figures and Tables

**Table 1:** The non-repudiation in each phase.

	Item	Signature Value	Sender	Receiver	Signature Verification
Phase					
Insurance purchasing phase		$\left(r_{PH-IC},s_{PH-IC}\right)$	PH	IC	$r_{PH-IC}\overset{?}{=}{x_{PH-IC}}^{\prime }\mathrm{mod}n$
		$(r_{IC-PH},$ $s_{IC-PH})$	IC	PH	$r_{IC-PH}\overset{?}{=}{x_{IC-PH}}^{\prime }\mathrm{mod}n$
Traffic accident phase		$\left(r_{PH-PA},s_{PH-PA}\right)$	PH	PA	$r_{PH-PA}\overset{?}{=}{x_{PH-PA}}^{\prime }\mathrm{mod}n$
		$\left(r_{PA-PH},s_{PA-PH}\right)$	PA	PH	$r_{PA-PH}\overset{?}{=}{x_{PA-PH}}^{\prime }\mathrm{mod}n$
Medical phase		$\left(r_{PH-MI},s_{PH-MI}\right)$	PH	MI	$r_{PH-MI}\overset{?}{=}{x_{PH-MI}}^{\prime }\mathrm{mod}n$
		$\left(r_{MI-PH},s_{MI-PH}\right)$	MI	PH	$r_{MI-PH}\overset{?}{=}{x_{MI-PH}}^{\prime }\mathrm{mod}n$
Request the traffic accident identification process		$\left(r_{IC-PA},s_{IC-PA}\right)$	IC	PA	$r_{IC-PA}\overset{?}{=}{x_{IC-PA}}^{\prime }\mathrm{mod}n$
		$\left(r_{PA-IC},s_{PA-IC}\right)$	PA	IC	$r_{PA-IC}\overset{?}{=}{x_{PA-IC}}^{\prime }\mathrm{mod}n$
Request the medical diagnostic information process		$\left(r_{IC-MI},s_{IC-MI}\right)$	IC	MI	$r_{IC-MI}\overset{?}{=}{x_{IC-MI}}^{\prime }\mathrm{mod}n$
		$\left(r_{MI-IC},s_{MI-IC}\right)$	MI	IC	$r_{MI-IC}\overset{?}{=}{x_{MI-IC}}^{\prime }\mathrm{mod}n$
Claims payment phase		$\left(r_{IC-BK},s_{IC-BK}\right)$	IC	BK	$r_{IC-BK}\overset{?}{=}{x_{IC-BK}}^{\prime }\mathrm{mod}n$
		$\left(r_{BK-PH},s_{BK-PH}\right)$	BK	PH	$r_{BK-PH}\overset{?}{=}{x_{BK-PH}}^{\prime }\mathrm{mod}n$

**Table 2:** Prevention of data manipulation in each phase.

	Item	Sender	Receiver	Encrypted Messages
Phase				
Insurance purchasing phase		PH	IC	$token=h(ID_{PH},{M_{\det ail}}^{\prime },TS_{PH-IC})$
		IC	PH	$token=h(ID_{PA},C_{IC-PH},TS_{IC-PH})$
Traffic accident phase		PH	PA	$token=h(ID_{PH},C_{PH-PA},TS_{PH-PA})$
		PA	PH	$token=h(ID_{PA},C_{PA-PH},TS_{PA-PH})$
Medical phase		PH	MI	$token=h(ID_{PH},C_{PH-MI},TS_{PH-MI})$
		MI	PH	$token=h(ID_{MI},C_{MI-PH},TS_{MI-PH})$
Request the traffic accident identification process		IC	PA	$token=h(ID_{IC},C_{IC-PA},TS_{IC-PA})$
		PA	IC	$token=h(ID_{PA},C_{PA-IC},TS_{PA-IC})$
Request the medical diagnostic information process		IC	MI	$token=h(ID_{IC},C_{IC-MI},TS_{IC-MI})$
		MI	IC	$token=h(ID_{MI},C_{MI-IC},TS_{MI-IC})$
Claims payment phase		IC	BK	$token=h(ID_{IC},C_{IC-BK},TS_{IC-BK})$
		BK	PH	$token=h(ID_{BK},C_{BK-PH},TS_{BK-PH})$

**Table 3:** The man-in-the-middle attack defense in each phase.

	Item	Sender	Receiver	Sender Encryption Use Public Key	Receiver Decryption Uses a Private Key
Phase					
Insurance purchasing phase		PH	IC	$C_{PH-IC}=E_{PUK_{IC}}({M_{\det ail}}^{\prime })$	${M_{\det ail}}^{\prime }=D_{PRK_{IC}}(C_{PH-IC})$
		IC	PH	$C_{IC-PH}=E_{PUK_{PH}}(M_{Contract})$	$M_{IC-PH}=D_{PRK_{PH}}(C_{IC-PH})$
Traffic accident phase		PH	PA	$C_{PH-PA}=E_{PUK_{PA}}(M_{PH-OS-PA})$	$M_{PH-OS-PA}=D_{PRK_{PA}}(C_{PH-PA})$
		PA	PH	$C_{PA-PH}=E_{PUK_{PH}}(M_{PA-PH})$	$M_{PA-PH}=D_{PRK_{PH}}(C_{PA-PH})$
Medical phase		PH	MI	$C_{PH-MI}=E_{PUK_{MI}}(M_{PH-MI})$	$M_{PH-MI}=D_{PRK_{MI}}(C_{PH-MI})$
		MI	PH	$C_{MI-PH}=E_{PUK_{PH}}(M_{MI-PH})$	$M_{MI-PH}=D_{PRK_{PH}}(C_{MI-PH})$
Request the traffic accident identification process		IC	PA	$C_{IC-PA}=E_{PUK_{PA}}(M_{IC-PA})$	$M_{IC-PA}=D_{PRK_{PA}}(C_{IC-PA})$
		PA	IC	$C_{PA-IC}=E_{PUK_{IC}}(M_{PA-IC})$	$M_{PA-IC}=D_{PRK_{IC}}(C_{PA-IC})$
Request the medical diagnostic information process		IC	MI	$C_{IC-MI}=E_{PUK_{MI}}(M_{IC-MI})$	$M_{IC-MI}=D_{PRK_{MI}}(C_{IC-MI})$
		MI	IC	$C_{MI-IC}=E_{PUK_{IC}}(M_{MI-IC})$	$M_{MI-IC}=D_{PRK_{IC}}(C_{MI-IC})$
Claims payment phase		IC	BK	$C_{IC-BK}=E_{PUK_{BK}}(M_{IC-BK})$	$M_{IC-BK}=D_{PRK_{BK}}(C_{IC-BK})$
		BK	PH	$C_{BK-PH}=E_{PUK_{PH}}(M_{BK-PH})$	$M_{BK-PH}=D_{PRK_{PH}}(C_{BK-PH})$

**Table 4:** Cost in time for each stage of computing.

	Party	PH	IC	PA	MI	BK
Phase						
Insurance purchasing phase		*2* $T_{asy}$ * + 2* $T_{amp}$ * + 3* $T_{h}$ * + 11* $T_{mul}$	*2* $T_{asy}$ * + 2* $T_{amp}$ * + 3* $T_{h}$ * + 11* $T_{mul}$	*N/A*	*N/A*	*N/A*
Traffic accident phase		*2* $T_{asy}$ * + 2* $T_{amp}$ * + 3* $T_{h}$ * + 11* $T_{mul}$	*N/A*	*2* $T_{asy}$ * + 2* $T_{amp}$ * + 3* $T_{h}$ * + 11* $T_{mul}$	*N/A*	*N/A*
Medical phase		*2* $T_{asy}$ * + 2* $T_{amp}$ * + 3* $T_{h}$ * + 11* $T_{mul}$	*N/A*	*N/A*	*2* $T_{asy}$ * + 2* $T_{amp}$ * + 3* $T_{h}$ * + 11* $T_{mul}$	*N/A*
Request the traffic accident identification process		*N/A*	*2* $T_{asy}$ * + 2* $T_{amp}$ * + 3* $T_{h}$ * + 11* $T_{mul}$	*2* $T_{asy}$ * + 2* $T_{amp}$ * + 3* $T_{h}$ * + 11* $T_{mul}$	*N/A*	*N/A*
Request the medical diagnostic information process		*N/A*	*2* $T_{asy}$ * + 2* $T_{amp}$ * + 3* $T_{h}$ * + 11* $T_{mul}$	*N/A*	*2* $T_{asy}$ * + 2* $T_{amp}$ * + 3* $T_{h}$ * + 11* $T_{mul}$	*N/A*
Claims payment phase		$T_{asy}$ * + 2* $T_{amp}$ * + * *2* $T_{h}$ * + 6* $T_{mul}$	*1* $T_{asy}$ * + 1* $T_{h}$ * + * *5* $T_{mul}$	*N/A*	*N/A*	*2* $T_{asy}$ * + 2* $T_{amp}$ * + 3* $T_{h}$ * + 11* $T_{mul}$

Notes: $T_{asy}$: Signature generation/validation calculation time. $T_{amp}$: Calculation time of the determination of the function. $T_{h}$: Hash calculation time. $T_{mul}$: Multiplication time.

**Table 5:** Communication cost for each stage.

	Item	Message Length	Rounds	4G	5G
Phase					
Insurance purchasing phase		4128 bits	2	0.04 ms	0.2 µs
Traffic accident phase		4128 bits	2	0.04 ms	0.2 µs
Medical phase		4128 bits	2	0.04 ms	0.2 µs
Request the traffic accident identification process		3392 bits	2	0.033 ms	0.17 µs
Request the medical diagnostic information process		3392 bits	2	0.033 ms	0.17 µs
Claims payment phase		3392 bits	2	0.033 ms	0.17 µs

**Table 6:** Compare other related articles.

Authors	Year	Objective	1	2	3	4	5	6
Dhieb et al. [23]	2020	A Secure AI-Driven Architecture for Automated Insurance Systems: Fraud Detection and Risk Measurement	Y	N	N	Y	Y	N
Jin et al. [24]	2020	Non-Repudiation Storage and Access Control Scheme of Insurance Data Based on Blockchain in IPFS	Y	N	Y	N	Y	Y
Zhe et al. [25]	2020	Blockchain and IoT for Insurance: A Case Study and Cyberinfrastructure Solution on Fine-Grained Transportation Insurance	Y	N	N	Y	N	N
Qi et al. [26]	2021	Scalable Decentralized Privacy-Preserving Usage-Based Insurance for Vehicles	Y	Y	N	N	Y	N
Alnuaimi et al. [27]	2022	Blockchain-Based Processing of Health Insurance Claims for Prescription Drugs	Y	N	Y	N	Y	Y
Ours	2022	A Blockchain and IPFS-Based Anticounterfeit Traceable of Car Insurance Claims System	Y	Y	Y	Y	Y	Y

Notes: 1: Blockchain focus, 2: Transportation-related systems, 3. IPFS focus, 4. Hyperledger focus, 5. Detailed protocols, 6. Security analysis, Y: Yes, and N: No.

## Data Availability

Data used to support the findings of this study are available from the corresponding author upon request.

## Abbreviations

$ID_{X}$	The identification number of the role X
$ID_{Policy}$	An identity of PH’s policy
$ID_{TA}$	An identity of traffic accident
$d_{X}$	Role X’s ECDSA private key
$Q_{X}$	Role X’s ECDSA public key
G	Generating points on elliptic curves
$k_{i}$	A random value on the elliptic curve
$\left(r_{x},s_{x}\right)$	The elliptic curve signature value of X
$\left(x_{x},y_{x}\right)$	Elliptic curve characteristic value of X
h(.)	Hash function
$BC_{A-B}$	Information that should be uploaded to the message sent by A to B
*OS*	The owner of the other party to the traffic accident
$M_{Policy}$	The PH’s signed insurance policy
$M_{A-B}$	Message sent from A to B
$M_{PH-OS-PA}$	Message submitted to the PA by the PH and the OS
$M_{Picture}$	Traffic accident-related picture information
$BK_{PH}$	PH’s bank account
$PIC_{PH}$	Hash values for PH’s traffic accident photos uploaded to IPFS
$IDE_{PH}$	Hash values for PH’s traffic accident identification uploaded to IPFS
$CERT_{PH}$	The hash values of the PH’s medical diagnosis uploaded to IPFS
$REC_{PH}$	The hash values of the PH’s medical receipt uploaded to IPFS
$CC_{PH}$	The hash values of the PH’s claim certificate uploaded to IPFS
$CD_{PH}$	Hash values of the PH’s claim detail uploaded to IPFS
$C_{A-B}$	The ciphertext of the information transmitted from A to B
$PUK_{X}$	The public key of the role X
$PRK_{X}$	The private key of the role X
$E_{PUK_{X}}(M)$	Encrypt *M* by $PUK_{X}$
$D_{PRK_{X}}(C)$	Decrypt *C* by $PRK_{X}$
$TS_{A-B}$	Timestamped messages sent from A to B
ΔT	Threshold of timestamp
$A\overset{?}{=}B$	Verify whether A is equal to B
